# The Probiotic *Lactobacillus paracasei* Ameliorates Diarrhea Cause by *Escherichia coli* O_8_ *via* Gut Microbiota Modulation^1^

**DOI:** 10.3389/fnut.2022.878808

**Published:** 2022-05-17

**Authors:** Shunan Ren, Chunjie Wang, Aorigele Chen, Wenting Lv, Ruijuan Gao

**Affiliations:** ^1^College of Veterinary Medicine, Inner Mongolia Agricultural University, Hohhot, China; ^2^College of Animal Science, Inner Mongolia Agricultural University, Hohhot, China

**Keywords:** koumiss, diarrhea, tight junction protein, IL-6, IL-1β, TNF-α, gut microbiome

## Abstract

**Introduction:**

Koumiss is a fermented horse milk food containing abundant probiotics. *Lactobacillus paracasei* is a bacterial strain isolated from koumiss that helps regulate the intestinal microbiota. One of the major cause of diarrhea is an imbalance of the intestinal flora. The aim of this study was to investigate whether *Lactobacillus paracasei* can ameliorate *E. coli*-induced diarrhea and modulate the gut microbiota.

**Methods:**

Mouse models of diarrhea were established *via* intragastric *E. coli* O_8_ administration. We then attempted to prevent or treat diarrhea in the mice *via* intragastric administration of a 3 × 10^8^ CFU/mL *L. paracasei* cell suspension. The severity of diarrhea was evaluated based on the body weight, diarrhea rate, and index, fecal diameter, ileum injury, hematoxylin-eosin (H&E) staining, and diamine oxidase (DAO) and zonulin expression. Expression of the tight junction (TJ) proteins claudin-1, occludin, and zona occludens (ZO-)1 were detected by immunohistochemistry (IHC). Gastrointestinal mRNA expression levels of interleukin (IL)-6, IL-1β, and tumor necrosis factor (TNF)-α were detected by real-time polymerase chain reaction (RT-PCR). The microbial composition was analyzed by 16s rRNA sequencing.

**Results:**

The *L. paracasei* demonstrated excellent therapeutic efficacy against diarrhea. It elevated the TJ protein levels and downregulated proinflammatory cytokines IL-6, IL-1β, TNF-α, and p65, myosin light chain 2 (MLC2), myosin light chain kinase (MLCK). Moreover *L. paracasei* increased those bacteria, which can product short-chain fatty acid (SCFA) such *Alistipes, Odoribacter, Roseburia*, and *Oscillibacter*.

**Conclusion:**

*L. paracasei* ameliorated diarrhea by inhibiting activation of the nuclear factor kappa B (NF-κB)-MLCK pathway and increasing the abundance of gut microbiota that produce SCFA.

## Introduction

Diarrhea caused by infection with enteropathogenic bacteria is a major global human health concern. According to the World Health Organization (WHO), about two billion cases of diarrhea caused by pathogenic bacteria occur annually. In developing countries, diarrhea by *Escherichia coli* (*E. coli*) is a common cause of childhood morbidity and mortality (1). *E. coli* is a gram-negative bacterium that causes severe diarrhea and rapid dehydration. *E. coli* cells adhere to the intestinal microvilli, cause lesions, secrete toxins (2), lower transepithelial electrical resistance (3), destroy tight junctions (TJs) (4), and dysregulate the intestinal microbiota.

Diamine oxidase (DAO) is an intracellular enzyme that catalyzes diamines in mammalian small intestinal epithelial cells. It can protect the intestinal mucosa by regulating ion balance in cells, affecting signal transduction pathways, and promoting cell repair (5). DAO is normally present in very small amounts in the circulation, its levels are correlated with the maturity and integrity of the intestinal mucosa (6), consequently, when the small intestine mucosal barrier function decreases or the intestinal epithelial cells die, the DAO enters the circulation, serum DAO levels are elevated, so, the DAO is an important biomarker for estimating the severity of intestinal mucosal disorders (7).

Zonulin is a protein that is synthesized in the intestine and regulates intestinal permeability (8). It opens TJs between cells, plays a pivotal role in TJ regulation during developmental, physiological, and pathological processes (9). Zonulin is normally absent in the circulation after the intestinal mucosal barrier is damaged the zonulin enters the circulation, therefore, zonulin also reflects the integrity of the intestinal mucosal barrier. Some pathogenic bacteria, such as *E. coli* is a factor that induces the release of Zonulin (10).

Intercellular TJs are dynamic structures involved in vectorial transport of water and electrolytes across the intestinal epithelium (9), the intestinal epithelium represents the largest interface between the external environment and the internal host milieu and constitutes the major barrier through which molecules can either be absorbed or secreted (11). There is now substantial evidence that TJs play a major role in regulating epithelial permeability by influencing paracellular flow of fluid and solutes (11). *E. coli* has a complex biological arsenal, it adheres to the surface of epithelial cells and induces accumulation of cytoskeletal proteins, destroy the TJs, and product pro-inflammatory cytokine (12), nuclear factor (NF)-κB controls the expression of essentially all pro-inflammatory cytokines (12). After *E. coli* infection, NF-κB is activated, and these pro-inflammatory factors such as interleukin (IL)-6, IL-1β, and tumor necrosis factor (TNF)-α lead TJ damage (13), while NF-κB activates myosin light chain kinase (MLCK) and then activates myosin light chain (MLC) (13), which makes IL-1β and TNF-α levels increased, and caused the contraction of peri-junctional actin-myosin filaments and opening of the barrier (14). Therefore, inhibition of NF-κB and MLCK activity can prevent increased intestinal permeability and TJ protein destruction.

A healthy host is not usually susceptible to infection by pathogenic microorganisms as the normal intestine contains abundant and diverse symbiotic microbial taxa. They prevent the invasion of microbial pathogens and help maintain a healthy micro-ecological balance (15). This anti-infectious capacity is referred to as “colonization resistance” (16). Gut microbiota continuously interacts with the vital organs of the host. Gut-kidney (15), gut-brain (17), and gut-bone marrow associations have been proposed, and prior research has already furnished ample empirical evidence for them.

Probiotics occur in the gastrointestinal tracts of various animal species. They help maintain micro-ecological balance and confer health benefits to the host. The concept of probiotics was first suggested by Elie Metchnikoff (18) who reported that the ingestion of certain bacteria may benefit the human gastrointestinal tract. In 2013, the Food and Agriculture Organization (FAO) of the United Nations/World Health Organization (UN/WHO) defined probiotics as live microorganisms that persist in the intestinal environment and have a salutary effect on health of the host when they are administered in sufficient quantities (19). Recently, probiotic getting more and more attention in health maintenance. It is now known that certain digestive disorders in humans are caused by deficiency or compromise of the intestinal microbiota. These include inflammatory bowel diseases (IBD) (20) such as ileitis and colitis (21) and travelers’ diarrhea (22). Probiotics can resist dysbiosis, enhance digestive health, prevent intestinal disease (23), alter the gut bacterial composition, upregulate TJ proteins, and prevent the penetration of microbial pathogens into the intestinal epithelium (21). They also regulate inflammatory (24) and immune response by modulating intestinal microbiota and stimulating immunocytes (25). Though they are isolated from the feces of healthy humans, probiotic strains also abound in fermented foods and those of animal origin (18). Koumiss is a traditional food make by fermented mare’s milk with locally occurring microorganisms (26) such as Lactic acid bacteria (LAB), which considered as candidate probiotics (27). It also reported to have favorable effect in human health (28, 29). *Lactobacillus paracasei* (*L. paracasei*) is a strain isolated from koumiss. It tolerates low pH, bile salts, simulated gastric secretions and environments, intestinal fluids, and enhanced jejunal TJ (30). In the present study, we administered *L. paracasei* to treat *E. coli-*induced diarrhea and used 16s rRNA analysis to investigate the effects of the probiotic on the gut microbiome. A major purpose of this work was to examine the prophylactic and therapeutic efficacy of *L. paracasei* against diarrhea.

## Experimental Procedures

### Bacterial Strains

The *L. paracasei* used in this study was isolated from koumiss made by the local herdsmen of Aluke’erqin qi, Inner Mongolia, China. *Escherichia coli* O_8_ (*E. coli* O_8_) isolated from the feces of calves presenting with diarrhea and was used to establish a mouse diarrhea model. All bacterial strains were stored at the College of Veterinary Medicine of Inner Mongolia Agricultural University. The *L. paracasei* was prepared from a 48-h static culture grown in deMan, Rogosa, and Sharpe (MRS) broth at 37°C. The *E. coli* O_8_ was prepared from a 12-h shake culture raised in nutrient broth at 37°C.

### Mice

Sixty male specific-pathogen-free Kunming mice (age 3–4 weeks, weight 18–22 g) were purchased from the Inner Mongolia Medical University Experimental Animal Center, Hohhot, China and housed in a sterile environment there (Product Number: SCXK2020-0001). The mice were maintained at an average temperature of 20°C ± 2°C; under a standard 12 h/12 h light/dark cycle (8:00 a.m. to 8:00 p.m.) and at 45% ± 10% relative humidity (RH). All mice were adaptively fed for 1 week before the experiments and had *ad libitum* access to a sterilized diet and water. Mouse feeding and the experimental procedures were performed in strict accordance with the provisions of the Experimental Care and Ethics Committee of Inner Mongolia Agricultural University (No. [2020]001).

### Mouse Models and Treatments

**FIGURE 1:**
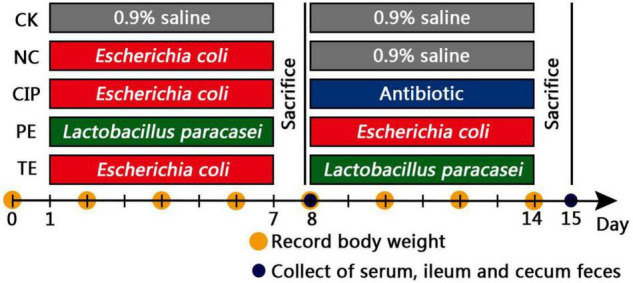
Treatment scheme of *L. paracasei* intervention.

The experimental scheme is shown in Figure 1. The mice had *ad libitum* food access and there was no difference among treatment groups in terms of food intake. The diarrhea models were established in the negative control (NC), antibiotic (CIP), and treatment (TE) groups by administering 0.2 mL/10 g *E. coli* O_8_ (3 × 10^13^ CFU/mL) for 1 week. The control group (CK) was intragastrically administered 0.9% (w/v) physiological saline on days 1–14. The NC group was administered *E. coli* O_8_ on days 1–7 and 0.9% (w/v) physiological saline on days 8–14. The CIP group was administered *E. coli* O_8_ on days 1–7 and 125 μg/ml ciprofloxacin on days 8–14. The pretreated (PE) mice were administered 0.2 mL/10 g probiotic *L. paracasei* (3 × 10^8^ CFU/mL) thrice daily for 1 week followed by *E. coli* O_8_ until day 14. The mice in the treatment (TE) group were administered *E. coli* O_8_ on days 1–7 and then 0.2 mL/10 g probiotic *L. paracasei* thrice daily on days 8–14. The mode of administration is intragastric administration.

### Sample Collection

The mice were separated in cages and the floors were covered with filter paper to facilitate observation of the fecal smudges. At days 0, 2, 4, 6, 8, 10, 12, and 14, mouse body weights were recorded. On days of 0, 3, 7, 10, and 14, the rates of diarrhea (1) were calculated. The diarrhea indices (2) were calculated based on the stool rates (3) and stool levels (4) and the fecal scores (Table 1).


diarrhea rate=diarrheamice/totalmiceineachgroup×100%(1)

diarrheaindex=stoolrate×stoollevel(2)

stool rate=thenumberofstoolsperanimal/totalstools×100%(3)

stool level=sumofallfecalscoring/numberoffecal(4)

**TABLE 1:** Fecal scoring.

Feces scoring				
**Score**	**1**	**2**	**3**	**4**
Fecal diameter (cm)	<1	1-1.9	2-3	>3

On days 8 and 15, six mice per group were sacrificed by intraperitoneal injection of 0.1% (w/v) pentobarbital sodium at a dose of 40 mg/kg. Postmortem examinations were then performed. The livers, spleens, lungs, and left and right kidneys were excised and weighed. The ilea were collected and sliced into two sections. One of these was stored in liquid nitrogen until western blot analysis and RT-PCR. The other was fixed in 4% (v/v) paraformaldehyde and used in histological and immunohistochemical (IHC) analyses. Blood samples were collected and the serum were separated and immediately stored at -80°C. The samples used to determine the cecal bacterial flora were collected and stored at -80°C until 16s rRNA sequencing analysis.

### Enzyme-Linked Immunosorbent Assay

The serum DAO and zonulin levels were measured by rat DAO and zonulin enzyme-linked immunosorbent assay (Elabscience, Bethesda, MD, United States). All procedures were performed according to the manufacturers’ instructions. The tests were performed in triplicate.

### Ileum Histopathology

The ilea were fixed in 4% (v/v) paraformaldehyde for 1 day and embedded in paraffin. Paraffin sections were prepared for 4 μm, dewaxed with water, stained with H&E, examined under a microscope, photographed, and scored according to Table 2.

**TABLE 2:** Histological scoring.

Score	Morphology
0	Normal mucosa, no inflammation
1	Mucosal goblet cell loss, low inflammatory infiltration
2	Mucosal goblet cell loss with medium inflammatory infiltration, enlarged inferior intestinal epithelial cell space
3	Mucosal recess absent, high level of inflammatory infiltration, increased vascular density
4	Mucosal goblet cells disappeared, high level of inflammatory infiltration, vascular density increased, enlarged inferior intestinal epithelial cell space

### Immunohistochemistry

The paraffin sections were recovered by heating to 98°C in 10 mM citrate buffer (pH 6.0) for 10 min. Endogenous peroxidase was blocked with 10% (v/v) H_2_O_2_ for 30 min. Non-specific antigens were blocked with serum at room temperature (20–25 °C) for 30 min. The paraffin sections were incubated with rabbit anti-occludin (1:100; DF7504; Affinity Biosciences Ltd., Liyang, China), rabbit anti-claudin-1 (1:100; AF0127; Affinity Biosciences Ltd., Liyang, China), and rabbit anti-ZO-1 (1:100; AF5145; Affinity Biosciences Ltd., Liyang, China) primary antibodies at 4 °C overnight and then treated with horseradish peroxidase (HRP)-conjugated goat anti-rabbit IgG secondary antibodies (1:200; GB23303; Wuhan Servicebio Technology Co., Ltd., Wuhan, China). Photographic images were acquired under an OLYMPUS microscope and analyzed with ImagePro Plus software (Media Cybernetics, Rockville, MD, United States).

### Quantitative Real-Time Polymerase Chain Reaction

Total RNA was extracted from the ilea with RNA extraction kit (AP-MN-MS-RNA-250; Axygen Biosciences; Union City, CA, United States) according to the kit manufacturer’s instructions was used to evaluated the expression levels of IL-6, IL-1β and TNF-α. Adjust the sample concentration to 400 μg/mL, then 500 ng total RNA was reverse-transcribed in 10 μL final volume with a PrimeScript RT Master Mix Kit (TaKaRa, RR036A, Japan), includes 5 × PrimeScript Master Mix 2 μL, total RNA 1.5 μL and RNase Free H_2_O 6.5 μL. Reverse transcription proceeded at 37°C for 15 min, 85°C for 5 s, and 4°C for 10 min. RT-qPCR was performed with SYBR Green Master Mix in an Applied Biosystems 7500 Fast Real-Time PCR system (Applied Biosystems, Foster City, CA, United States). Then 100 ng total RNA amount of DNA template in 20 μL final volume with a Real-Time PCR Kit (TaKaRa, RR820A, Japan), the 20μL reaction system includes TB Green *Premix Ex Taq* II (Tli RNaseH Plus) 10 μL, PCR Forward Primer 0.8 μL, PCR Reverse Primer 0.8 μL, ROX Reference Dye II 0.4 μL, cDNA 2 μL, Nuclease-Free water 6 μL. The PCR program consisted of an initial denaturation step at 95°C for 30 s followed by 40 cycles of 95°C for 3 s and 60°C for 30 s. Relative mRNA expression was analyzed by the 2^–ΔΔ^ *^Ct^* method and normalized to β-actin expression levels. The primers used in the RT-PCR are listed in Table 3, all primers were synthesized by Sangon Biotech, ShangHai, China.

**TABLE 3:** The information of primer sequence designed.

ID	Gene	Primer sequence
NM_031168.2	IL-6	F: CTTCTTGGGACTGATGCTGGTGAC
		R: AGTGGTATCCTCTGTGAAGTCTCCTC
NM_008361.4	IL-1β	F: CACTACAGGCTCCGAGATGAACAAC
		R: TGTCGTTGCTTGGTTCTCCTTGTAC
NM_001278601.1	TNF-α	F: CGCTCTTCTGTCTACTGAACTTCGG
		R: GTGGTTTGTGAGTGTGAGGGTCTG
NM_007393.5	β-actin	F: GTGCTATGTTGCTCTAGACTTCG
		R: ATGCCACAGGATTCCATACC

### Western Blotting

The total protein was extracted from ilea and then quantitated using BCA Protein Assay kit. Equal amounts of protein (20 μg) from different samples were separated by 6∼15% SDS-PAGE and transferred to PVDF membranes. The membranes were blocked with 5% skimmed milk for 4 h at room temperature, then probed with ZO-1 (1:1,000, AF5145, Affinity), claudin-1 (1:1,000, AF0127, Affinity), occludin (1:1,000, DF7504, Affinity), p65 (1:1,000, AF5002, Affinity), MLCK (1:1,000, AF5314, Affinity), MLC2 (1:1,000, 36725, CST), and β-actin (1:3,000, ab8226, Abcam) was used as loading control, overnight at 4°C and with secondary antibody at RT for 1 h. The images were captured using ChemiDoc MP imaging system (Bio-Rad).

### Intestinal Flora 16S rRNA Gene Sequencing

Cecal contents were collected from 4 mice per group at days 8 and 15, for microbiome analysis. Microbial genomic DNA was extracted from fecal samples using the E.Z.N.A. soil DNA Kit (Omega Bio-Tek, Norcross, GA, United States) according to the manufacturer’s instructions. The DNA extract was checked on 1% agarose gel, and DNA concentration and purity were determined with NanoDrop 2000 UV-vis spectrophotometer (Thermo Fisher Scientific, Wilmington, United States). The hypervariable region V3-V4 of the bacterial 16S rRNA gene were amplified with primer pairs 338F (5′-ACTCCTACGGGAGGCAGCAG-3′) and 806R (5′-GGACTACHVGGGTWTCTAAT-3′) by an ABI GeneAmp 9700 PCR thermocycler (ABI, CA, United States). The PCR amplification of 16S rRNA gene was performed as follows: initial denaturation at 95°C for 3 min, followed by 27 cycles of denaturing at 95°C for 30 s, annealing at 55°C for 30 s and extension at 72°C for 45 s, and single extension at 72°C for 10 min, and end at 4°C. The PCR mixtures contain 5 × TransStart FastPfu buffer 4 μL, 2.5 mM dNTPs 2 μL, forward primer (5 μM) 0.8 μL, reverse primer (5 μM) 0.8 μL, TransStart FastPfu DNA Polymerase 0.4 μL, template DNA 10 ng, and finally ddH_2_O up to 20 μL. PCR reactions were performed in triplicate. The PCR product was extracted from 2% agarose gel and purified using the AxyPrep DNA Gel Extraction Kit (Axygen Biosciences, Union City, CA, United States) according to manufacturer’s instructions and quantified using Quantus Fluorometer (Promega, United States). Purified amplicons were pooled in equimolar and paired-end sequenced on an Illumina MiSeq PE300 platform (Illumina, San Diego, United States) according to the standard protocols by Majorbio Bio-Pharm Technology (Shanghai, China). The raw 16S rRNA gene sequencing reads were demultiplexed, quality-filtered by fastp version 0.20.0 and merged by FLASH version 1.2.7 with the following criteria: (i) the 300 bp reads were truncated at any site receiving an average quality score of < 20 over a 50 bp sliding window, and the truncated reads shorter than 50 bp were discarded, reads containing ambiguous characters were also discarded; (ii) only overlapping sequences longer than 10 bp were assembled according to their overlapped sequence. The maximum mismatch ratio of overlap region is 0.2. Reads that could not be assembled were discarded; (iii) Samples were distinguished according to the barcode and primers, and the sequence direction was adjusted, exact barcode matching, 2 nucleotide mismatches in primer matching. Operational taxonomic units (OTUs) with 97% similarity cutoff were clustered using UPARSE version 7.1, and chimeric sequences were identified and removed. The taxonomy of each OTU representative sequence was analyzed by RDP Classifier version 2.2 against the 16S rRNA database (Silva v138) using confidence threshold of 0.7.

### Statistical Analysis

Statistical significance analyzed using the software GraphPad Prism. Student’s *t*-test to determine the levels of significance for comparison between two groups and statistical significance among more than two groups was calculated using one-way ANOVA with Tukey’s test. *P*-values of less than 0.05 were considered significant (*P < 0.05; ^**^P < 0.01; ^***^P < 0.001; ^****^P < 0.0001; “ns,” no significant). The results were reported as mean ± *SD*. Spearman’s correlation between the bacteria and the diarrhea-related parameters was conducted using the programming language R (version 4.0.2).

## Results

### *Lactobacillus paracasei* Administration Attenuated Diarrhea

**FIGURE 2:**
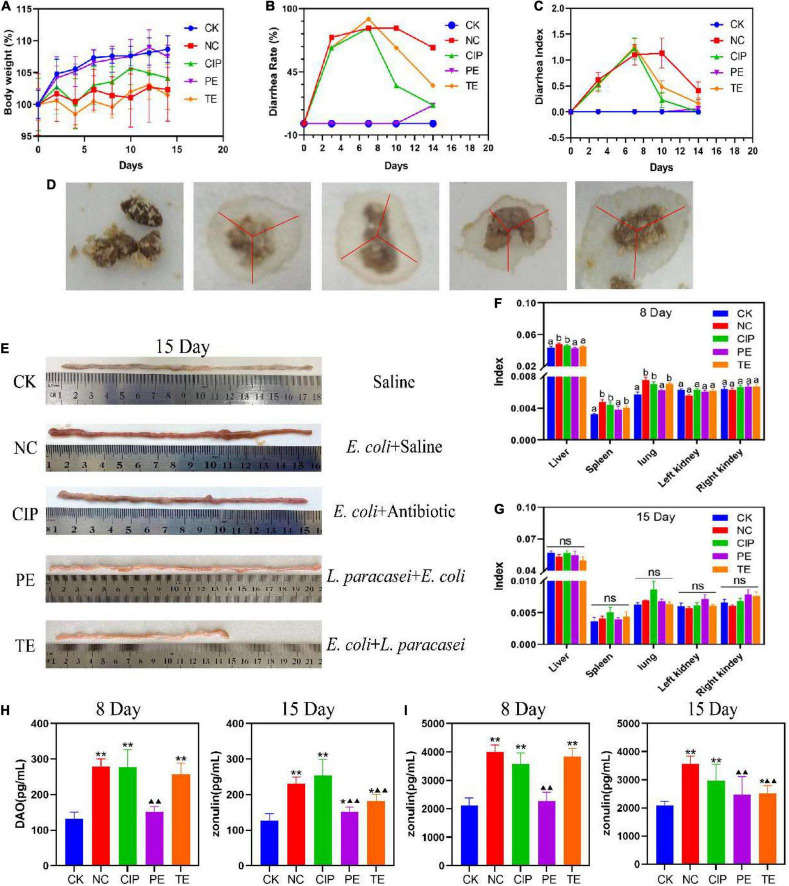
Changes in the parameters of mice during the administration with *L. paracasei*. **(A)** Body weight. **(B)** Diarrhea rate. **(C)** Diarrhea index. **(D)** Fecal diameter. **(E)** Ileum injury. **(F,H)** the organ index at the days of 8 and 15. Different letters in each figure indicates significant in the data at *P* < 0.05. **(H,I)** Representative the expression of DAO and zonulin. * Represent compare with CK group. *P < 0.05; **P < 0.01; ^▲^represent compare with NC group. ^▲^*P* < 0.05; ^▲▲^*P* < 0.01. Calculated by Student’s *t*-test. CK, the normal group; NC, the negative group; CIP, the diarrhea model group treated with ciprofloxacin; PE, the diarrhea model group prevented with *L. paracasei*; TE, the diarrhea model group treated with *L. paracasei.*

Compared with CK group, the groups administered *E. coli* O_8_ presented with significant reductions in body weight (Figure 2A), and increased diarrhea rates (Figure 2B) and indices (Figure 2C). Hence, the mouse diarrhea model was successfully established. The *E. coli* administrate caused diarrhea (Figure 2D) followed by intestinal congestion and edema (Figure 2E). Compared with the NC group, the PE group showed significant increase in body weight and decreases in the diarrhea rates and indices. Thus, the *L. paracasei* treatment helped prevent *E. coli* O_8_-induced diarrhea. Compared with the NC group, the TE group presented with lower diarrhea rates and indices but no significant change in body weight after *L. paracasei* administration. On day 8, the NC, CIP, and TE groups presented with significantly lower spleen and lung indices than the CK and PE groups (Figure 2F). By day 15, there were no significant differences among groups in terms of their liver, spleen, lung, left kidney, and right kidney indices (Figure 2G). Therefore, *L. paracasei* efficiently treated *E. coli* O_8_-induced diarrhea.

DAO is a highly active structural enzyme in the intestinal epithelium (31) and its blood levels reflect intestinal barrier integrity. Figure 2H shows that on day 8, the DAO level was significantly higher in the *E. coli*-treated group than the CK group (*P* < 0.01) but there were no significant differences between the PE and CK groups (*P* > 0.05) in terms of DAO level. On day 15, in the TE group DAO significantly downregulated (*P* < 0.01). The blood DAO levels were higher for the CIP than the NC group (*P* < 0.01). After *E. coli* intervention, the DAO content of the PE group was significantly lower than those of the TE and NC groups (*P* < 0.01) but higher than that of the CK group (*P* < 0.05).

Zonulin is the main regulator of the epithelial TJ and, by extension, the intestinal barrier. Figure 2I shows that the *E. coli* treatment significantly upregulated the serum zonulin level (*P* < 0.01). On day 15, the zonulin content was significantly lower in the PE and TE groups than the NC group after *L. paracasei* intervention (*P* < 0.01). However, the zonulin content was still higher in the TE group than the CK group (*P* < 0.05). The zonulin content was still significantly higher in the CIP group than the CK group (*P* < 0.01).

In addition, we also found the levels of DAO and Zonulin in the NC group on day 15 were still higher than those in the PE group, although this value was lower than the value after 7 days of *E. coli* administrate, it also seemed to indicate that *E. coli* would affect intestinal permeability for a longer time, while the content of DAO and Zonulin in *L. paracasei* increased after the intervention was changed to *E. coli* challenge, but it was not significant compared with the CK group, which indicated that *L. paracasei* had a certain protective effect on intestinal damage caused by *E. coli*, and long-term intervention of *L. paracasei* may have more profound benefits on the body’s intestinal barrier.

**FIGURE 3:**
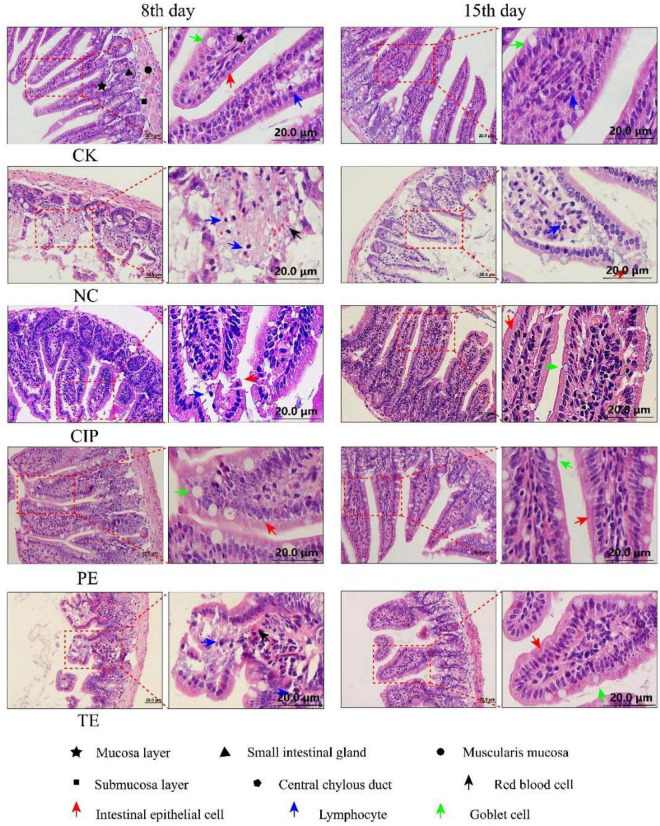
Representative H&E staining microscopic images of ileum tissues from different groups. CK, the normal group; NC, the negative group; CIP, the diarrhea model group treated with ciprofloxacin; PE, the diarrhea model group prevented with *L. paracasei*; TE, the diarrhea model group treated with *L. paracasei.*

**FIGURE 4:**
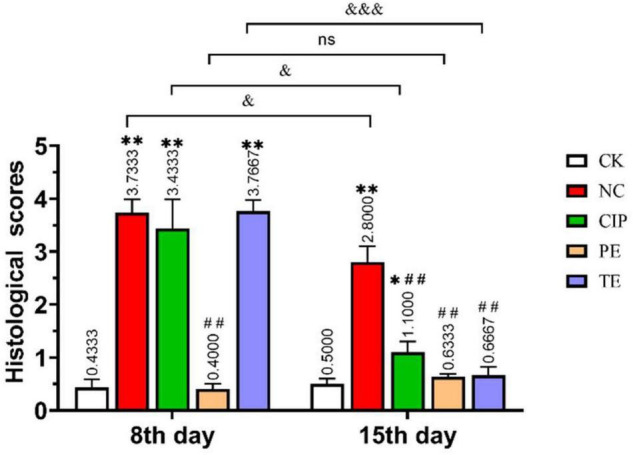
Histological injury score of the ileum in mice. *Represent compare with CK group. *P < 0.05; ^**^P < 0.01; # represent compare with NC group. ## *P* < 0.01; & represent compare with after therapy. & *P* < 0.05; &&& *P* < 0.001.

H&E staining of the ileal tissue (Figure 3) disclosed that *E. coli* O_8_ administration damaged the intestinal villi and increased the numbers of lymphocytes in the lamina propria and epithelium. In contrast, prophylactic or therapeutic *L. paracasei* administration restored the intestinal villi. Hence, *L. paracasei* administration alleviated the *E. coli* O_8_-induced diarrhea pathogenesis. Although *L. paracasei* could prevent diarrhea, on day 15, the intestinal villi were nonetheless slightly damaged after *E. coli* administration. This finding suggests that *L. paracasei* may require long-term administration for optimal efficacy (Figure 4).

In histological morphology observation, after 15 days of intervention in each group, the ileal length of the CK group was 17.5 cm, NC group was 15.6 cm, CIP group was 15 cm, PE group was 20.5 cm, and TE group was 15 cm. There was no significant growth after *L. paracasei* treatment, but comparable to the NC and CIP groups, the intestinal tissue was not swollen. In the HE staining observation, we observed a very small amount of inflammatory infiltration in the TE group and observed intact intestinal villi with more goblet cells, which was consistent with the ileal morphology of the TE group in Figure 2E.

### *Lactobacillus paracasei* Enhance Tight Junction Proteins Expression

**FIGURE 5:**
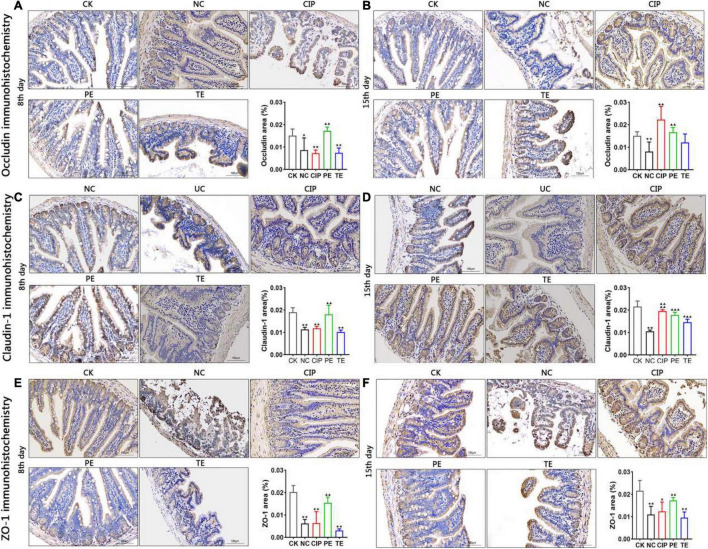
TJ proteins expression with *E. coli* and *L. paracasei*. **(A,C,E)** Representative the occludin, claudin-1 and ZO-1 protein repressive at day 8. CK group administrate with 0.9% saline; PE group administrate with *L. paracasei*; NC, CIP and TE groups administrate with *E. coli*. **(B,D,F)** Representative the occludin, claudin-1 and ZO-1 protein repressive at day 15. CK and NC group administrate with 0.9% saline; CIP group administrate with ciprofloxacin; PE group administrate with *E. coli*, and TE group administrate with *L. paracasei*. *Representative compared with CK group. *P < 0.05; **P < 0.01. ^▲^Representative compared with NC group. ^▲▲^*P* < 0.01, ^▲▲▲^*P* < 0.001, ^▲▲▲▲^*P* < 0.0001 as determined by Student’s *t*-test.

**FIGURE 6:**
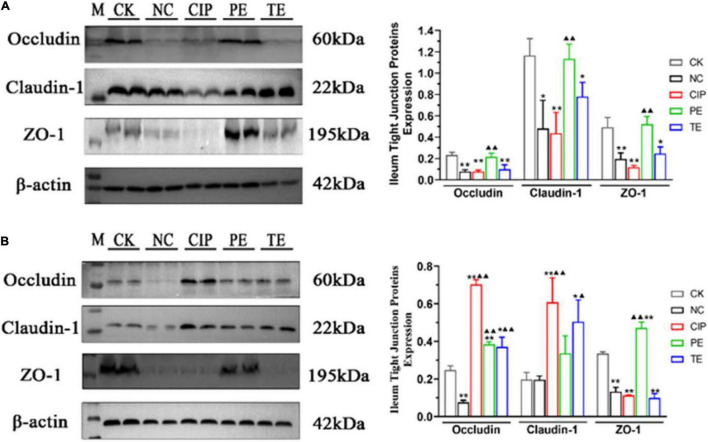
Protein expression levels of occludin, claudin-1, ZO-1 on days 8 **(A)** and 15 **(B)**. “M” representative Protein Marker; *Compared with CK group. *P < 0.05; **P < 0.01; ^▲^Compared with NC group. ^▲^*P* < 0.05; ^▲▲^*P* < 0.01 as determined by Student’s *t*-test.

Immunohistochemistry (IHC) (Figure 5) and western blotting (WB) (Figure 6) were used to investigate the roles of L. paracasei in diarrhea and determine whether its probiotic action depends on TJ protein expression. Figures 5A,C,E, shows IHC staining of the ileum tissue indicated that *E. coli* treatment groups NC, CIP, and TE presented with occludin, claudin-1 and ZO-1 protein downregulation. These effects were largely reversed in response to prophylactic *L. paracasei* administration. This treatment kept the TJ proteins and the ileal architecture intact (Figures 5B,D,F). WB confirmed that *E. coli* downregulated the TJ protein whereas *L. paracasei* alleviated the damage *E. coli* caused to the TJ (Figure 6A). The antibiotic therapy also upregulated the TJ Proteins (Figure 6B). Therefore, *L. paracasei* administration reinforced the TJ which, in turn, played an important role in the antidiarrheal efficacy of the probiotic.

### Oral *Lactobacillus paracasei* Attenuates Diarrhea and Inhibition NF-κB-MLCK Pathway After *Escherichia coli* O_8_ Administrate

**FIGURE 7:**
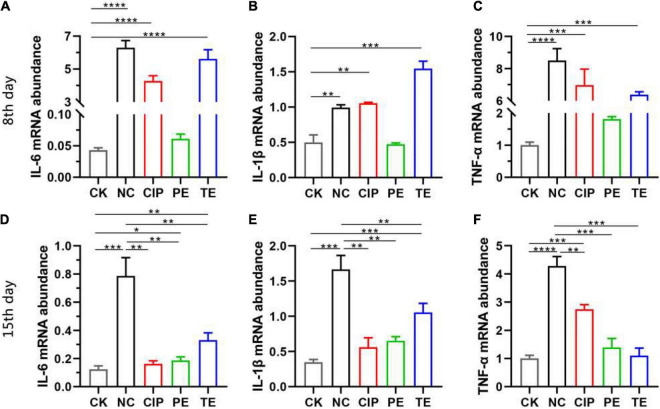
The probiotic *L. paracasei* inhibit inflammation induced by diarrhea. **(A–C)** Representative the expression of IL-6, IL-1β and TNF-α in ileum at day 8 after *E. coli* O_8_ determined using RT-PCR in mice. **(D–F)** Representative the expression of IL-6, IL-1β and TNF-α in ileum at day 15 after ciprofloxacin, *E. coli* O8 and *L. paracasei* administrated using RT-PCR in mice. *P < 0.05, **P < 0.01, ***P < 0.001, ****P < 0.0001 as determined by Student’s *t*-test.

To explore the effects of *L. paracasei* on diarrhea, we orally administered *L. paracasei* or saline to the mice thrice daily for 1 week before or after they were subjected to *E. coli* O_8_-induced injury. Using RT-PCR, we identified the relative differences in ileal IL-6, IL-1β, and TNF-α expression. As shown in Figure 7 *E. coli* administrated upregulated IL-6, IL-1β, and TNF-α in the NC, CIP, and TE group (Figures 7A–C), *L. paracasei* treatment before *E. coli* O_8_ administration did not upregulate IL-6, IL-1β, or TNF-α compared with the CK group (*P* > 0.05), however, *L. paracasei* treatment after *E. coli* O_8_ administration significantly downregulated IL-6, IL-1β, and TNF-α in the TE group compared with the NC group (*P* < 0.01). There were no significant differences between the CIP and NC groups in terms of their IL-6, IL-1β, and TNF-α expression levels (*P* > 0.05) (Figures 7D–F).

**FIGURE 8:**
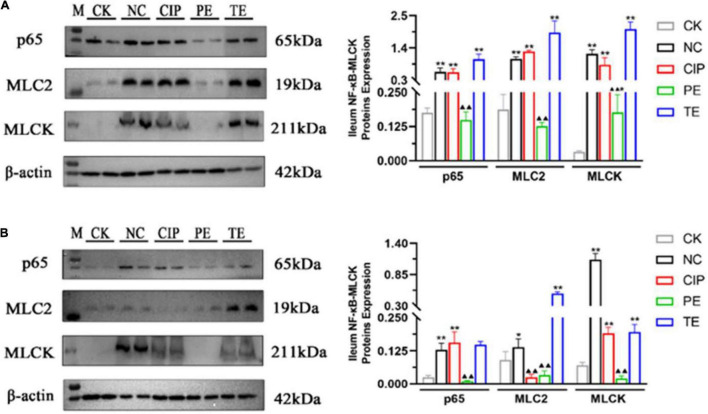
Protein expression levels of IκBα, p65, MLC2, MLCK and β-actin on days 8 **(A)** and 15 **(B)**. *Representative compared with CK group. *P < 0.05, **P < 0.01. ^▲^Representative compared with NC group. ^▲^*P* < 0.05, ^▲▲^*P* < 0.01 as determined by Student’s *t*-test.

We used WB to analyze the NF-κB-MLCK pathway and determine whether the observed changes in IL-6, IL-1β, and TNF-α were associated with the NF-κB pathway (Figures 8A,B). The *E. coli* administration activated the NF-κB-MLCK pathway and upregulated NF-κB, p65, MLC2, and MLCK. The *L. paracasei* administration did not upregulate p65, MLC2, or MLCK (Figure 8A). After *L. paracasei* therapy, though, p65, MLC2, and MLCK were downregulated but there were no significant decreases in their expression levels in either the TE or the CK group (Figure 8B). Thus, *L. paracasei* administration was more efficacious prophylactically than therapeutically.

### The Change of Probiotic-Induced Gut Microbiotic Ameliorates *Escherichia coli* Induced Diarrhea

**FIGURE 9:**
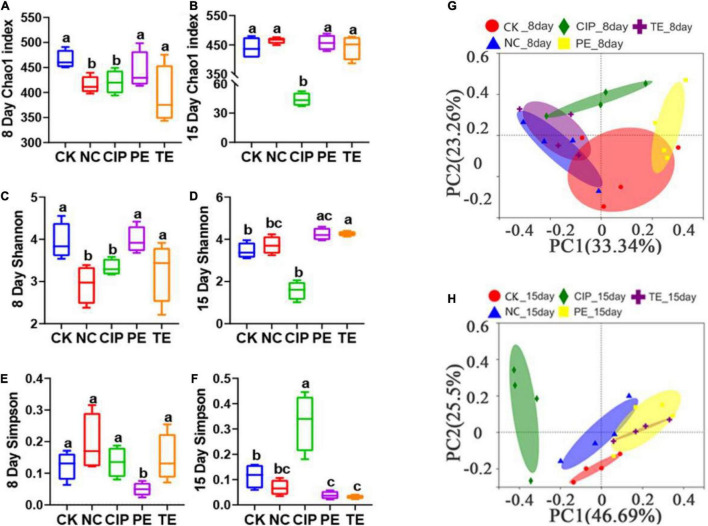
Gut microbiota diversity. **(A,B)** Alpha diversity boxplot of the Chao1 index on day 8 and 15. **(C,D)** Alpha diversity boxplot of the Shannon index on day 8 and 15. **(E,F)** Alpha diversity boxplot of Simpson index on day 8 and 15. **(G,H)** PCA using unweight-unifrac of beta diversity on day 8 and 15. The sane small-letter means no different *P* > 0.05; Different lowercase letters have significant differences *P* < 0.05.

A 16S rRNA analysis was performed on the mouse cecal contents to investigate the intestinal microbiota after *L. paracasei* administration. Figures 9A,C,D shows that the Chao1 and Shannon indices indicated significant differences between the *E. coli* O8-administered groups and the CK and PE groups in terms of their intestinal microbiota α-diversity (Figures 9A,C). However, the PE group had a significantly lower Simpson index than the other groups (Figure 9E). The Shannon and Simpson indices were significantly increased in response to *L. paracasei* administration (Figures 9D,F), but there were no significant differences among the CK, NC, PE, and TE groups in terms of these parameters. The CIP group presented with significantly lower Chao1 and Shannon indices than the other groups (Figures 9B,D). The Simpson indices significantly increased following ciprofloxacin administration (Figure 9F).

We performed a principal coordinate analysis (PCoA) based on a weighted UniFrac distance to analyze the β-diversity of the gut microbial composition. We found significant differences between the *E. coli*-administered and CK groups in terms of their gut microbiome β-diversity. The diarrhea model mice pretreated with *L. paracasei* and the CK group differed in terms of their β-diversity (Figure 9G). On day 15, the β-diversity of the groups administered *L. paracasei* was similar to that of the CK group. β-diversity was similarly elevated for both the PE and TE groups. β-diversity was significantly different between the group administered ciprofloxacin and the CK group (Figure 9H).

**FIGURE 10:**
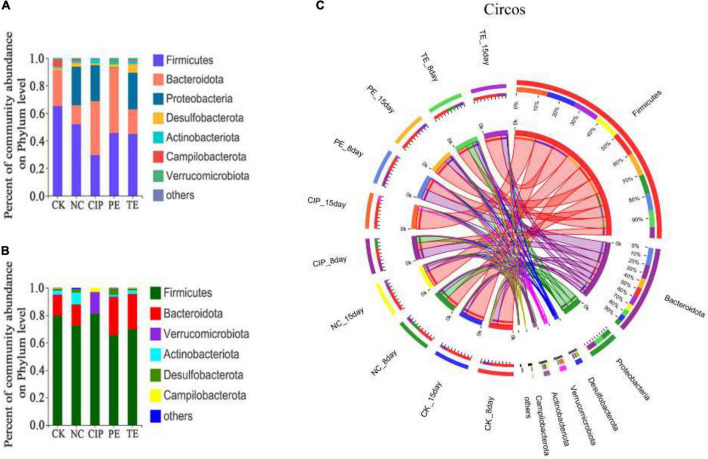
Percent of community abundance on phylum level. **(A,B)** Bar plots of the taxonomic composition at the phylum on day 8 and 15. **(C)** Circus analysis.

The potential compositions of the gut microbiota differed among the five groups. At the phylum level, all of them presented with similar taxonomic communities and relatively high abundances of Firmicutes and Bacteroidota. Compared with CK and PE groups, the NC, CIP, and PE group exhibited significantly reduced Bacteroidota and increased Proteobacteria and Firmicutes/Bacteroidetes ratios (Figure 10A). These changes were reversed in the *E. coli* O_8_-administered groups treated with *L. paracasei* and ciprofloxacin (Figure 10B). Ciprofloxacin administration lowered relative gut microbiota diversity and increased the abundances of Firmicutes, Bacteroidota, Verrucomicrobiota, and Campylobacteriota (Figure 10C).

**FIGURE 11:**
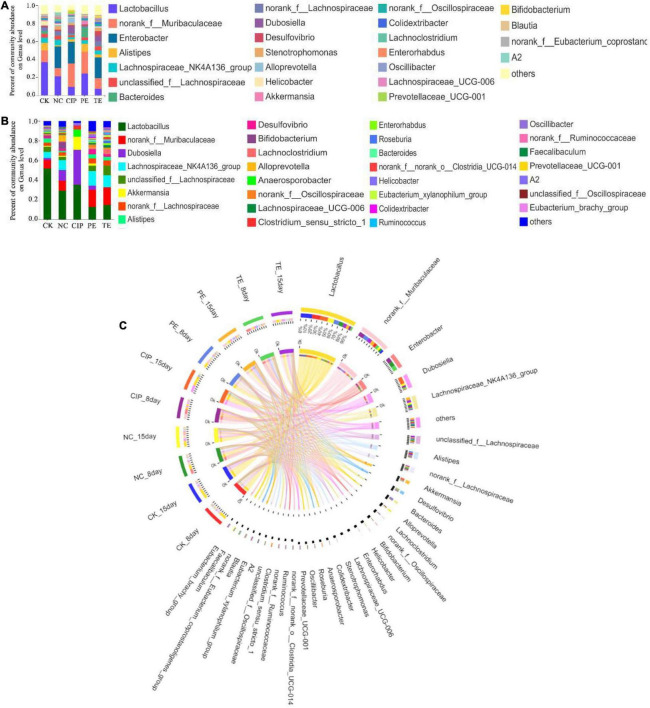
Percent of community abundance on genus level. **(A,B)** Bar plots of the taxonomic composition at the genus on day 8 and 15. **(C)** Circus analysis.

We also analyzed the taxonomic communities at the genus level (Figures 11A,B). Enterobacter significantly increased after *E. coli* O_8_ administration. Oral *L. paracasei* administration decreased *Enterobacter* and increased *Lactobacillus*. The gut microbiota comprised *Lactobacillus*, *Dubosiella*, *Akkermansia*, and *Anaerosporobacter* after ciprofloxacin administration (Figure 11C).

### Correlation Between Intestinal Flora and Diarrhea Indexes

A heatmap was plotted for Spearman’s correlations among gut microbiota abundance and the diarrhea-related indices (body weights, diarrhea rates and indices, liver, spleen, lung, and left and right kidney indices, DAO, zonulin, occludin, claudin-1, ZO-1, IL-6, IL-1β, and TNF-α).

**FIGURE 12:**
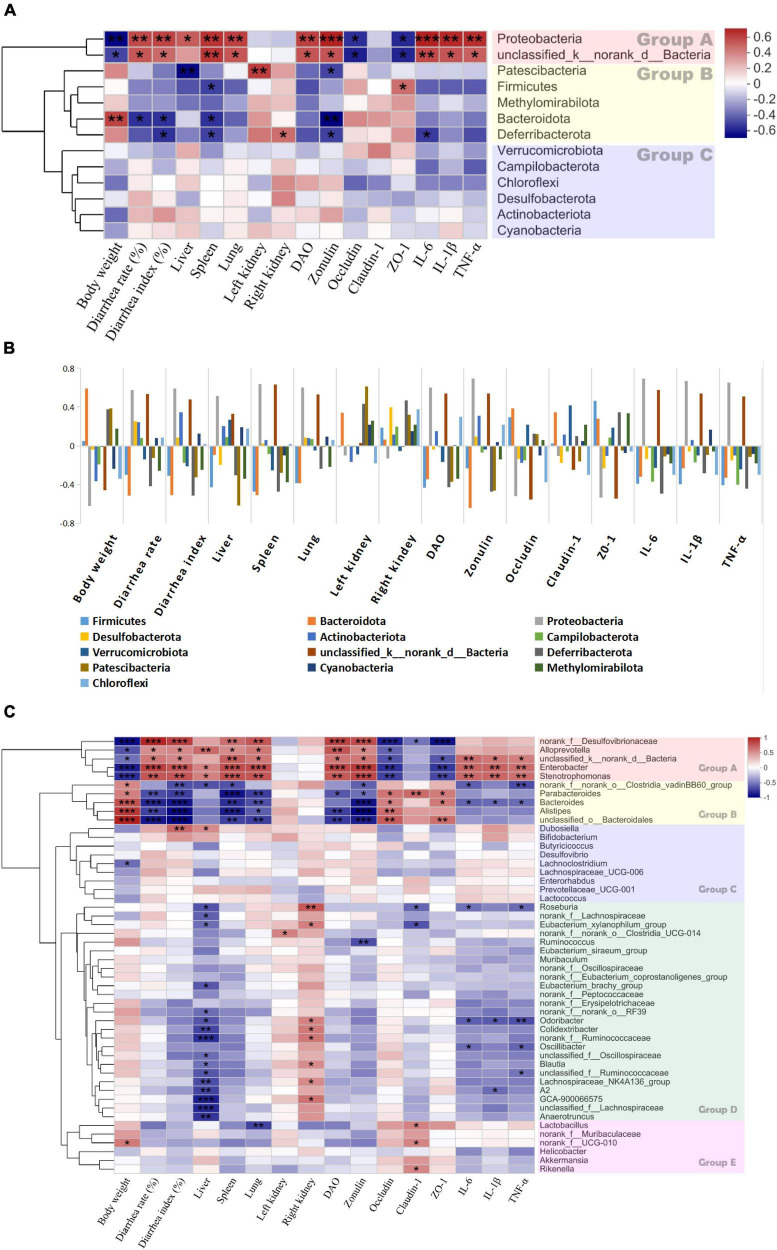
Heatmap of spearman’s correlation between the bacteria and diarrhea-related parameters. **(A)** The correlation between bacterial phylum and diarrhea-related indexes. **(B)** The correlation between bacterial phylum and diarrhea-related indexes with histogram. **(C)** The correlation between bacterial genera and diarrhea-related indexes on day 8. Significant difference determined at *P ≤ 0.05; **P ≤ 0.01; ***P ≤ 0.001. Groups A, B, C, D and E are formed based on the clustering results of the bacterial.

The clustering analysis in Figure 12 shows that the bacterial phyla were clearly divided into three groups (Figure 12A). The phyla in group A were negatively correlated with body weight and the TJ proteins but positively correlated with the diarrhea rates and indices, the organ indices, DAO, zonulin, and the proinflammatory factors. Bacterial abundance was significantly increased in the *E. coli* treatment group (Figure 11A). Therefore, an increase in any of these phyla could promote diarrhea. Conversely, the phyla in group B were positively correlated with body weight and the TJ proteins but negatively correlated with the diarrhea rates and indices, the organ indices, DAO, zonulin, and the proinflammatory factors (Figures 11A,B). Therefore, an increase in any of these phyla could inhibit diarrhea.

Similar phenomena were also observed at the genus level. Figure 12C shows that the genera were divided into five groups. Those in group A were positively correlated with body weight and TJ proteins but negatively correlated with the diarrhea rate and indices, DAO, zonulin, and the proinflammatory factors. The genera in group B were not significantly correlated with the diarrhea-related indices. The genera in group C were positively correlated with the diarrhea rates and proinflammatory factors but not significantly correlated with the diarrhea-related indices. The abundances of these genera were correlated with IL-6, IL-1β, and TNF-α. The genera in group D were negatively correlated with the TJ proteins and the proinflammatory factors but positively correlated with the liver and spleen indices. The genera in group E were positively correlated with the diarrhea rates and indices, DAO, zonulin, and the proinflammatory factors but negatively correlated with body weight, the liver index, and the TJ proteins. Thus, any increase in these genera has therapeutic efficacy against diarrhea.

## Discussion

In the present study, *L. paracasei*, a strain isolated from koumiss, was first used to treat diarrhea caused by *E. coli* O_8_ in mice. The *L. paracasei* demonstrated superior preventative efficacy against the *E. coli* O_8_-induced diarrhea. It increased the relative body weight and decreased the diarrhea rate, diarrhea index, and the concentration of serum DAO and Zonulin in diarrhea mice, besides, *L. paracasei* also upregulation TJs protein content and improved relative ileal integrity.

Many strains have been reported to decrease intestinal permeability by downregulation of DAO and Zonulin. Intestinal mucosa damage can increase the Zonulin and DAO activity (32), and probiotics such as *Bifidobacterium*, *Lactobacillus* and *Streptococcus* (32), *Bacillus coagulans*, and *Lactobacillus plantarum* (7) can decrease the concentration of DAO and Zonulin, has been shown to be optimal for reducing inflammatory responses and bacterial translocation (33), LAB and *L. casei* DN-114 001 also reduced small bowel permeability in D-IBS (34, 35). In this study, *E. coli* O8 increased the DAO and Zonulin content induced intestinal permeability increase, *L. paracasei* administrated prevent this damage decreased the levels of DAO, Zonulin and improve the integrity of ileal.

TJs are the most apical organelles of the epithelial junction complexes. They are vital to epithelial barrier formation (34) and function and control paracellular pathway permeability (36). We examined ZO-1, claudin-1, and occludin distribution and expression and observed the effects of *L. paracasei* on them. *E. coli* adhered to the intestinal cells and damaged TJs structure. In this study, ZO-1, claudin-1, and occludin were significantly downregulated in the presence of *E. coli* compared with the control group. In contrast, both *L. paracasei* and ciprofloxacin improved intestinal barrier function. These changes were correlated with the IL-6, IL-1β, and TNF-α expression levels. Recent studies showed that IL-1β, IL-6, and TNF-α increase intestinal TJ permeability and induce TJ damage. They also play important roles in promoting intestinal inflammation (13). Previous studies showed that MLCK plays a central role in regulating intestinal TJ permeability. It activates the myosin light chain to induce cytoskeleton contraction and regulate the TJ (37). MLCK regulates ZO-1 (38–40), mediates claudin-1, activates occluding, and regulates the TJ structure. A recent study demonstrated that upregulation of IL-1β and TNF-α increases TJ permeability by inducing MLCK and redistributing the TJ (13, 41). TNF-α also mediates MLCK by inducing the NF-κB pathway (37). In the present study, *E. coli* administration upregulated IL-6, IL-1β, TNF-α, NF-κB p65, MLCK, and MLC2. We showed that *L. paracasei* downregulated IL-6, IL-1β, TNF-α, NF-κB p65, MLCK, and MLC2 and shortened the duration of *E. coli*-induced diarrhea. Hence, *L. paracasei* inhibits diarrhea by modulating the NF-κB-MLCK pathway. Another report indicated that Probio-M8 had anti-inflammatory efficacy (42). Based on our findings, we speculated that diarrhea prevention by *L. paracasei* is associated with the inhibition of NF-κB-MLCK upregulation and a decrease in the dysregulation of the epithelial barrier. Nevertheless, these mechanisms remain to be empirically validated.

We assessed the effects of intestinal microbiota dysbiosis and probiotics on *E. coli* O_8_-induced diarrhea progression. Intestinal microflora dysbiosis is the key event mediating intestinal inflammation and diarrhea. Pathogen infection altered the gut microbe composition by increasing the numbers of Proteobacteria and decreasing the abundances of Firmicutes and Bacteroidota. Here, ciprofloxacin treatment restored the intestinal barrier but also decreased intestinal microbial diversity and could paradoxically induce diarrhea. Compared with ciprofloxacin, administration of the probiotic *L. paracasei* more effectively enhanced intestinal microbial diversity. Prior reports showed that several single strains have been used to treat various diseases by regulating the intestinal microbiota. *Bifidobacterium lactis Probio*-M8 prevented and treated acute respiratory tract infections and shortened the duration of nasal, pharyngeal, and flu-like symptoms (42). *Bifidobacterium bifidum* G9-1 improved gastrointestinal symptoms in type 2 diabetes mellitus (T2DM) (43). Lab4 supplementation effectively prevented certain cardiovascular diseases (44). A combination of *L. paracasei* GMNL-89 plus *L. reuteri* GMNL-89 was administered as an adjuvant to control cancer progression, lower serum liver enzyme levels, and improve patient chemotherapy tolerance (45). Another study showed that a mixture of probiotic *Lactobacilli* and *Bifidobacteria* alleviated atopic dermatitis by modulating the gut microbiota (46). Compared with ciprofloxacin, probiotic *L. paracasei* improved intestinal barrier function, inhibited pathogen invasion, and increased intestinal microbial diversity. In contrast, ciprofloxacin does not distinguish between normal microbiota and harmful bacteria. In fact, antibiotic overuse may lead to antibiotic resistance and gut dysbiosis. The latter is associated with pathogen infection, irritable bowel syndrome (IBS), and IBD (47).

A change in the gut microbiota mediated by *L. paracasei* administration is vital to the efficacious treatment of *E. coli*-induced diarrhea. The gut microbiome differs between patients with diarrhea and healthy individuals. Diarrhea significantly lowers gut microbial α- and β-diversity and intestinal microbiota abundance. It also alters gut bacterial composition including the levels and ratios of harmful and probiotic bacteria (48). In diseased states, dysbiosis occurs when the harmful microbiota overtake the beneficial ones (49–52). Earlier studies showed that the *Proteobacteria* and *Enterobacter* (53, 54) and *Alloprevotella* (55, 56) were increased in digestive diseases. Certain bacteria such as *Roseburia* (57) ferment carbohydrates, lower pH, and promote butyrate biosynthesis. Abundant *Roseburia* can increase the gut SCFA content. SCFAs are metabolites that could potentially treat diarrhea (58, 59). Other bacteria such as *Akkermansia* and *Blautia* (60) can alleviate damage to the intestinal barrier damage caused by ulcerative colitis (UC) and inhibit the inflammatory response. *Clostridia* (61) significant decrease in patients with *Clostridium difficile*-positive diarrhea. *Parabacteroides* (62), *Alistipes* and *Odoribacter* (63) modulate metabolism and produce SCFAs. Here, *L. paracasei* administration increased the abundance of SCFA-producing bacteria including *Oscillibacter* (64), *Roseburia*, *Odoribacter*, and *Alistipea*.

**FIGURE 13:**
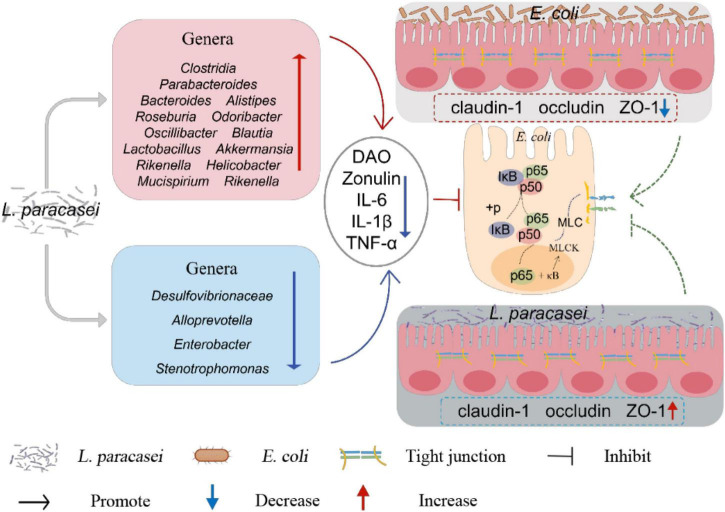
Overall mechanisms of *L. paracasei* for the treatment of diarrhea.

In general, in this study, *L. paracasei* administration was more efficacious prophylactically than therapeutically. *L. paracasei* increased the body weight and decreased the diarrhea rate, diarrhea index and the concentration of DAO and Zonulin in diarrhea mice, besides, *L. paracasei* also upregulating the expression of TJs proteins and downregulates the production of pro-inflammatory cytokines, inhibits activation of the NF-κB-MLCK signaling pathway. It also altered the structure of the intestinal microbiota, modifications to the structure of the intestinal microbiota may increase the abundance of SCFA-producing bacteria (Figure 13). These key factors may contribute to the therapeutic efficacy of *L. paracasei* and its ability to protect the intestinal barrier. However, to make it available in the clinic, further studies are needed to confirm these preliminary *in vivo* data.

## Data Availability Statement

The datasets presented in this study can be found in online repositories. The names of the repository/repositories and accession number(s) can be found below: NCBI Sequence Read Archive accession number: SRP363657 available at: https://trace.ncbi.nlm.nih.gov/Traces/sra/?study=SRP363657.

## Ethics Statement

The animal study was reviewed and approved by the Experimental Care and Ethics Committee of Inner Mongolia Agricultural University.

## Author Contributions

SR: conceptualization, methodology, data curation, formal analysis, writing, and original draft. CW: writing-review and editing, project administration, and funding acquisition. AC: supervision. WL: resources, investigation, and validation. RG: resources. All authors contributed to the article and approved the submitted version.

## Conflict of Interest

The authors declare that the research was conducted in the absence of any commercial or financial relationships that could be construed as a potential conflict of interest.

## Publisher’s Note

All claims expressed in this article are solely those of the authors and do not necessarily represent those of their affiliated organizations, or those of the publisher, the editors and the reviewers. Any product that may be evaluated in this article, or claim that may be made by its manufacturer, is not guaranteed or endorsed by the publisher.
